# Supercritical CO_2_ Foaming of Thermoplastic Materials Derived from Maize: Proof-of-Concept Use in Mammalian Cell Culture Applications

**DOI:** 10.1371/journal.pone.0122489

**Published:** 2015-04-10

**Authors:** Grissel Trujillo-de Santiago, Cynthia Guadalupe Portales-Cabrera, Roberto Portillo-Lara, Diana Araiz-Hernández, Maria Cristina Del Barone, Erika García-López, Cecilia Rojas-de Gante, María de los Angeles De Santiago-Miramontes, Juan Carlos Segoviano-Ramírez, Silverio García-Lara, Ciro Ángel Rodríguez-González, Mario Moisés Alvarez, Ernesto Di Maio, Salvatore Iannace

**Affiliations:** 1 Centro de Biotecnología-FEMSA, Tecnológico de Monterrey, Monterrey, Nuevo León, México; 2 Harvard-MIT Helath Sciences and Technology, Brigham and Women’s Hospital, Cambridge, Massachusetts, United States of America; 3 Institute of Polymers, Composites and Biomaterials, Consiglio Nazionale delle Ricerche, Naples, Italy; 4 Centro de Innovación en Diseño y Tecnología, Tecnológico de Monterrey, Monterrey, Nuevo León, México; 5 Departamento de Producción Animal, Universidad Autónoma Agraria Antonio Narro, Torreón, Coahuila, México; 6 Unidad de Bio-imagen/Microscopía, Universidad Autónoma de Nuevo León, Monterrey, Nuevo León, México; 7 Dipartimento di Ingegneria Chimica, dei Materiali e della Produzione Industriale, University of Naples Federico II, Naples, Italy; University of Akron, UNITED STATES

## Abstract

**Background:**

Foams are high porosity and low density materials. In nature, they are a common architecture. Some of their relevant technological applications include heat and sound insulation, lightweight materials, and tissue engineering scaffolds. Foams derived from natural polymers are particularly attractive for tissue culture due to their biodegradability and bio-compatibility. Here, the foaming potential of an extensive list of materials was assayed, including slabs elaborated from whole flour, the starch component only, or the protein fraction only of maize seeds.

**Methodology/Principal Findings:**

We used supercritical CO_2_ to produce foams from thermoplasticized maize derived materials. Polyethylene-glycol, sorbitol/glycerol, or urea/formamide were used as plasticizers. We report expansion ratios, porosities, average pore sizes, pore morphologies, and pore size distributions for these materials. High porosity foams were obtained from zein thermoplasticized with polyethylene glycol, and from starch thermoplasticized with urea/formamide. Zein foams had a higher porosity than starch foams (88% and 85%, respectively) and a narrower and more evenly distributed pore size. Starch foams exhibited a wider span of pore sizes and a larger average pore size than zein (208.84 vs. 55.43 μm^2^, respectively). Proof-of-concept cell culture experiments confirmed that mouse fibroblasts (NIH 3T3) and two different prostate cancer cell lines (22RV1, DU145) attached to and proliferated on zein foams.

**Conclusions/Significance:**

We conducted screening and proof-of-concept experiments on the fabrication of foams from cereal-based bioplastics. We propose that a key indicator of foamability is the strain at break of the materials to be foamed (as calculated from stress vs. strain rate curves). Zein foams exhibit attractive properties (average pore size, pore size distribution, and porosity) for cell culture applications; we were able to establish and sustain mammalian cell cultures on zein foams for extended time periods.

## Introduction

Foams, which can be defined as materials with high porosities at the micro-scale (and therefore low densities), represent a ubiquitous architecture in nature. Remarkable examples are tissues such as the cancellous or trabecular bones that contain marrow [1], the cavernous tissue of the mammalian penis [2], the pomelo peel of some citrus fruits [3], and the bodies of sea sponges [4]. Many applications are possible for synthetic foams, and they have been recently suggested as scaffolds for cell culture and tissue engineering [5–9]. Other applications include acoustic [10] and heat insulation [11–13], weight reduction [13], fabrication of flotation devices [14], etc.

Polyurethane foams have been used commercially for decades. However, the search for biodegradable substitutes for these petroleum-based foams is a current technological trend. Specifically, in the context of tissue engineering (and cell culture applications), foams derived from natural polymers are particularly attractive due to their distinctive characteristics such as bio-compatibility and biodegradability. Methods of fabrication and studies of characterization of foams derived from several biodegradable materials have been recently reported. These materials include poly-lactic acid (PLA) [15,16,17], cassava starch reinforced with microbial cellulose [18], poly(ε-caprolactone) (PCL) [15,19], poly(hydroxybutyrate-co-hydroxyvalerate) (PHBV) [20], poly(glycerol-sebacate) (PGS), poly(lactic acid) (PLA)/poly(ε-caprolactone) (PCL) blend, and poly(lactic-co-glycolic acid) (PLGA) [21], chitosan–polyester [22], collagen/hyaluronic acid/gelatin [6], gelatin/chitosan [23], gelatin [24], and zein, the maize protein [8,9,24–26], and poly (ethylene glycol)-co-(l-lactic acid) [27], among others.

Several different techniques have been used to produce foams, ranging from the classical method of salt leaching [21,23,28] to freeze-drying [23,29] and the use of microwave heating [13]. A common technique currently used to produce foams is gas foaming, consisting of the solubilization of a gaseous blowing agent into a molten thermoplastic polymer, under pressure, to form a molten polymer/gas solution. The subsequent release of pressure, usually during a thermo extrusion process, triggers gas evolution from the supersaturated solution and bubble nucleation and growth, finally creating the foam, which is then consolidated by solidification. [14]. In this work, we use a variation of this process, where supercritical CO_2_ is used as the foaming agent in a controlled batch autoclave [15,16,17,24,30].

Previous studies [25,31–33] have demonstrated that all relevant quality parameters of a foam (density, porosity, mean pore size, pore size distribution) are determined by the foaming process conditions, namely the blowing agent, (CO_2,_ N_2_, etc.), the temperature and pressure during gas solubilization, the pressure release profile during foaming, the cooling program after foaming, etc. Each material is characterized by a specific foaming processing window and specific optimum conditions that produce foams suitable for specific applications. The foaming potential (foamability) of a material is an intrinsic property of the material and is determined primarily by the interface, rheological, and thermal properties and the mass transport and sorption of the available blowing agents [17,34]. In natural polymers (i.e., polysaccharides and proteins), thermoplasticization has to be used in order to modify the structure of the material (typically, the tertiary and quaternary structures) to render it suitable for further processing and to confer plastic-like properties. In this case, thermoplasticization conditions and the eventual use of plasticizers also play a major role in determining the foamability of the material [35,36].

Nature-derived materials ultimately introduce an intrinsic variability (due to differences in sources, culture, harvest, extraction, etc.), which implies a necessity for adaptation of processing conditions to the specific materials [35]. Here, we have chosen the same set of conservative foaming conditions for maize-derived plastics obtained from different thermo-extrusion procedures; the supercritical foaming conditions selected have been used previously for a wide variety of bioplastics [24,33,37] and can be considered useful for assessing foaming potential.

Maize is an important and abundant natural resource in Central and North America. Thus far, only a few groups have reported the use of cereal-based materials to produce foams. Peng et al. [38] and Castillejo et al. [39] used wheat and corn starch, respectively. The elaboration and characterization of foams from zein, the main protein of maize, have also been reported [25,26,28,34,40]. In this investigation, our particular interest was (a) to identify thermoplasticization conditions suitable for the production of maize-derived foams; (b) to identify foaming predictive properties of maize-derived thermoplastics; and (c) to explore a set of strategies to improve the zein-starch compatibility previously identified as a problem [41,42] in the production of thermoplastic materials directly from whole maize flour. In addition, (d) we used simple techniques to characterize the produced foams in terms of macroscopic parameters (thickness expansion, surface area expansion, and volume expansion) and microstructure (overall porosity, pore size distribution, and pore morphology), and (e) conducted proof-of-principle experiments to determine the feasibility of use of zein materials for adhered cell culture applications.

To our knowledge, this is the first report that explores the whole path from thermoplasticization and foaming to cell culture. Here, we describe experiments on thermoplasticization, foaming, and cell culture using different materials derived from maize, highlighting the critical stages of this path in the selection of appropriate conditions (material, plasticizer, thermoplasticization, and foaming) for creation of a material that supports successful cell growth.

## Materials and Methods

### Raw materials

Whole blue maize flour, chemically modified maize flour, native and chemically modified maize starch, modified maize flour, and zein (the main protein in maize) were used in this study. Native maize starch was purchased from ALMEX (Guadalajara, México). Maize zein (code Z3625) and all plasticizers were purchased from Sigma-Aldrich. The plasticizers used were mixtures of urea-formamide at 2:1 ratio and sorbitol-glycerol at 1.4:1 (these have been reported to be adequate plasticizers for starch [35,43]) and polyethylene glycol 400 (PEG400) for zein [44]. All plasticizers were purchased from Sigma-Aldrich. Moisture content was determined for all starches and zein by TGA (TGA Q5000, TA Instruments, USA) in order to prepare the formulation blends on a dry basis. Blue maize (BM) was kindly provided by Eduardo Lovera (at Federación de Agricultores del Edomex). Flour (150 mesh, decorticated blue maize) was obtained according to Rojas de Gante et al. [45].

**Fig 1:**
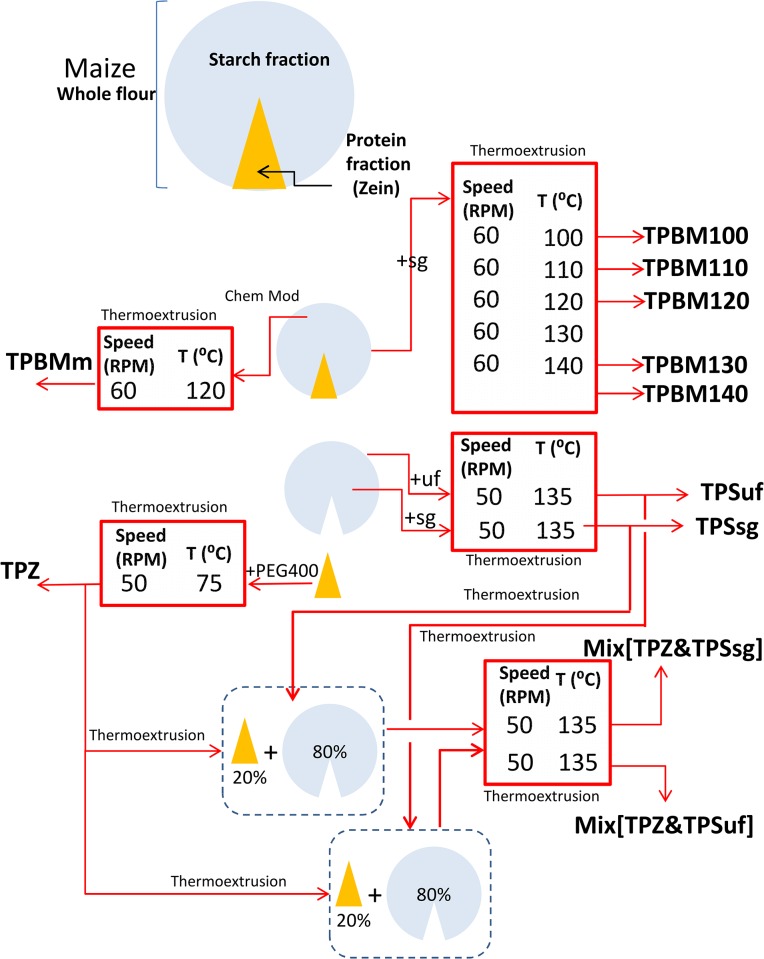
Schematic representation of the experimental treatments and materials derived from them: TPZ: thermoplasticized zein; TPS: thermoplasticized starch; TPBMx: thermoplasticized blue maize (x is a suffix that indicates extrusion temperature); TPmBM: thermoplasticized chemically modified blue maize (as described in materials and methods); Mix[TPS_y_+TPZ]^a^: thermoplasticized blends of TPS and TPZ (80:20 wt/wt). The y subindex indicates the plasticizer used to produce TPS. ^a^Blends were produced using the close mode compounding described in materials and methods. Plasticizers used where sg (sorbitol-glycerol); uf (urea-formamide); and PEG400 (poly-ethylene glycol with m.w. = 400 Da).

Modified starch and flour were prepared as described by Murúa-Pagola et al. [46]. Briefly, a dispersion of 585 g of hydrolyzed starch (prepared by acid hydrolysis in 3.4% HCl at 50°C and shaking at 500 rpm for 6 h) or BM or WS flour in 1.3 L of distilled water was prepared by shaking at 500 rpm. The pH was maintained at 8.5–9.0 using 1 M NaOH. About 2 g of maleic anhydride/50 g of starch (dry basis) were slowly added over 2 h. Agitation was increased as needed, reaching 1000 rpm when the slurry was very viscous. The reaction was run for 6 h and was stopped by lowering the pH to 4.8–5.2 using 1 M HCl. The modified starch was centrifuged for 20 min at 4500 rpm. The precipitate was washed-centrifuged three times and dried for 24 h in a convection oven at 45°C. The dried modified starch was milled and sieved to obtain a 150 mesh powder. The degree of substitution of the modified starch was 0.039±0.002 as determined by the volumetric method reported elsewhere [46]. Native starch, modified starch, zein, and the plasticizers were premixed with a spatula to provide a crude blend. Water content was determined for all starches and zein through TGA (TGA Q5000, TA Instruments, USA) in order to prepare the formulation blend on dry basis. The moisture content of starch, zein, and flour compositions was adjusted to 10% (dry basis) by addition of distilled water. Sorbitol/glycerol (1.4:1 wt/wt) and urea/formamide (2:1 wt/wt) and PEG 400 at 25% were used as plasticizers. Details of the compositions are reported in Fig 1 and Table 1.

**Table 1:** Processing conditions used to elaborate maize derived bioplastics later exposed to CO_2_ supercritical foaming conditions.

Biopolymer	Plasticizer(wt % plasticizer)	Speed of rotation (rpm)	Temperature(°C)
TPZ	PEG400 (25)	50	75
TPSuf	Uf (30)	50	135
TPSsg	sg (30)	50	135
Mix[TPSuf+TPZ]^a^	uf (30) for TPS & PEG400 (25) for TPZ	50	135
Mix[TPSsg+TPZ]^a^	sg (30) for TPS & PEG400 (25) for TPZ	50	135
TPBM140	sg (30)	60	140
TPBM130	sg (30)	60	130
TPBM120	sg (30)	60	120
TPBM110	sg (30)	60	110
TPBM100	sg (30)	60	100
TPmBM	sg (30)	60	120

**Plasticizers:** Peg400: polyethylene-glycol 400; uf: urea/formamide; sg: sorbitol and glycerol mixture (1.4:1 wt/wt).

**Bioplastics:** TPZ: thermoplasticized zein; TPS: thermoplasticized starch; TPBMx: thermoplasticized blue maize (x is a suffix that indicate extrusion temperature); TPmBM: thermoplasticized chemically modified blue maize (as described in materials and methods); Mix[TPS_y_+TPZ]^a^: thermoplasticized Blends of TPS and TPZ (80:20 wt/wt). The y subindex indicates the plasticizer used to produce TPS. ^a^Blends were produced using the close mode compounding described in materials and methods.

### Thermoextrusion and compression molding

**Fig 2:**
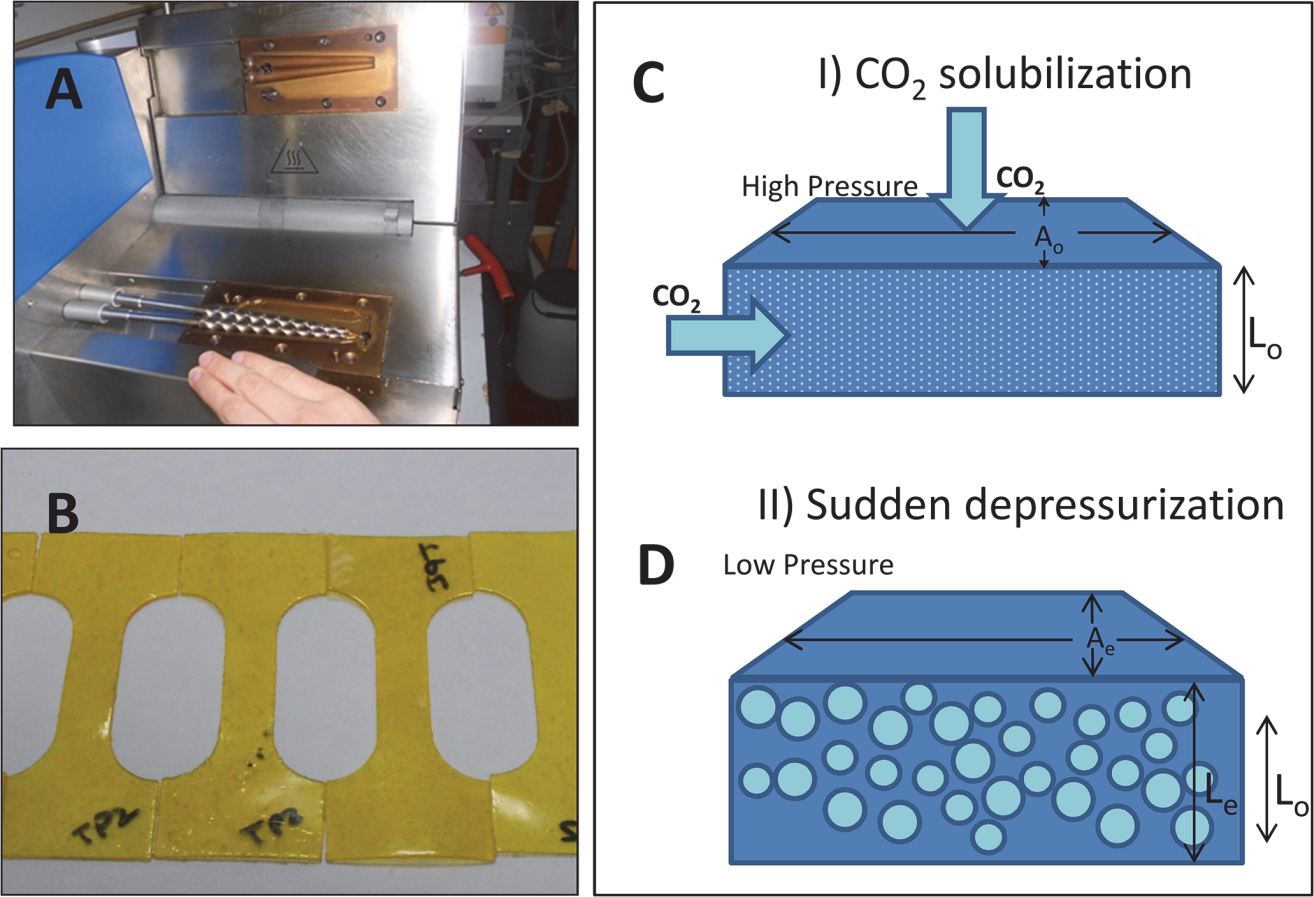
Maize-derived thermoplastic were produced by (A) thermo-extrusion in a twin conical screw mini extruder (Haake MiniLab, Thermo Scientific, USA) followed by thermopressing at 20 MPa for 4 min in a P300P press (Collin, Germany). (B) Slabs of these plastics were subjected to supercritical CO_2_ foaming, which occurred in two stages: (C) diffusion and solubilization of CO_2_ molecules within a solid material matrix at supercritical conditions, and (D) a sudden drop in pressure allows the formation of CO_2_ bubbles within the material.

Thermoplasticization of blends was performed in a twin conical screw mini extruder (Haake MiniLab, Thermo Scientific, USA; Fig 2A). Preliminary experiments were conducted to find suitable processing conditions for each composition, starting from values reported for similar materials [35,36,44,47]. Details on the mixing temperature and speed of rotation of screws are reported in Table 1. Extrusion was conducted either in open or closed mode. The open mode consisted of slow and continuous feeding of about 30 g of material into the miniextruder. This was done manually, assisted by an iron rod to press the material into the barrel, and the extruded material was continuously collected at the die outlet. This process lasted for about 24 to 28 min, from the very first amount fed into the mini-compounder to the collection of the last extruded material. The closed mode, which was used to process TPS-TPZ blends, consisted of manually feeding 7 g of the crude blend (for zein and starch slabs) or a mixture of pellets (for TPS-TPZ blends) into the mini-compounder over 5±0.25 minutes, mixing (with recirculation) the sample for three additional minutes, and then opening the mini compounder to collect the sample. Each process was conducted at least three times in order to ensure reproducibility. The thermoplasticized materials were pressed at 20 MPa for 4 min in a P300P press (Collin, Germany) at the same temperature used for extrusion and were then cooled to 35°C under pressure to obtained thermoplastic slabs (Fig 2B).

### Tensile testing of materials

The tensile strength and elongation at break of selected slabs obtained from maize based materials were evaluated using standard microtensile test specimens according to the ASTM D1708. Mechanical testing was performed on slabs (Fig 2B) conditioned at room temperature (about 25°C) and 50% RH prior to testing with a universal testing machine (Tensile T2020, Alpha Technologies, USA).

### Foaming

Foaming experiments were carried out on selected slabs according to a previously reported method [37] with slight modifications. Samples were placed into an autoclave (HiP, USA) equipped with a system of valves for CO_2_ injection and pressure and heating control. For most samples, the autoclave was closed, heated, and pressurized with CO_2_ until a set point of 15 MPa and 70°C was reached. Samples were kept at this condition for 4 h to solubilize CO_2_ into the materials until equilibrium (Fig 2C). Afterwards, the autoclave was cooled to 45°C. At this temperature, the pressure was released at a rate of 70 MPa/s, allowing sample foaming (Fig 2D). Foams were then immediately removed from the vessel. These foaming conditions gave the results reported in Table 2. To obtain wpTPZ foams, which were zein foams with a wider pore size (average pore size≈70 μm), a different set of conditions was used: thermo-plasticized zein slabs were heated and pressurized with CO_2_ until a set point of 10 MPa and 75°C was reached, samples were kept at this condition for 4 h, the autoclave was cooled to 45°C, and the pressure was released at a rate of 42 MPa/s.

**Table 2:** Relevant indicators of the quality of foams obtained by supercritical CO_2_ expansion in slabs derived from thermoplasticized starch plasticized with urea/formamide (TPSuf foams) and thermoplasticized zein (TPZ foams).

**Indicator**	**TPZ foams**	**TPSuf foams**
2D Porosity (as fraction of empty space)	0.625	0.442
Average pore size (μm^2^) ^1^	55.43	208.84
Standard deviation (μm^2^)	39.42	214.90
Median of pore size (μm^2^)	46.67	114.98
50% of pores below (μm^2^)	47	150
75% of pores below (μm^2^)	80	305
95% of pores below (μm^2^)	150	606
Percentage of pores above 200 μm^2^ (%)	0%	40%
Thickness expansion/√Surface expansion ^2^	1.81	1.50

^1^Pore size is expressed as the projected area of the pore as determined by image analysis of electronic microscope micrographs.

^2^Higher ratios of Thickness/Surface expansion indicate more spherical pores.

### Characterization of foams

The thickness and surface area of the slab samples were measured before and after CO_2_ treatment. Thickness was measured using a high precision digital caliper (Mitutoyo, Japan). Surface area was determined by image analysis using Image J software (NIH, USA). These data were used to calculate the volume and the expansion fold in volume. Volume was calculated as the product of the surface area times the slab thickness. Samples were weighed on an analytical balance (Mettler AE240, Mettler Toledo, Highstown, USA). Foam porosity was determined according to Eq. 1.

$$\text{porosity = }\left[\text{1-}\left({\text{ρ}}_{\text{a}}{\text{/ρ}}_{\text{b}}\right)\right]$$ 1

where ρ_a_ and ρ_b_ are the apparent densities of the materials after and before foaming, respectively, calculated as the mass to volume ratio of the samples.

The microstructure of the foams was analyzed by cross-sectioning with a razor blade and viewing by scanning electronic microscopy (SEM). Samples were vacuum metallized with gold-palladium by a Baltec MED 020 Coating System and analyzed by SEM using a FEI Quanta 200 FEG microscope (Eindhoven, The Netherlands).

The pore size distribution of selected foams (TPZ foams and TPSuf foams) was determined from SEM micrographs by image analysis using Image J. Briefly, one of the software tools was used to draw irregular close polygons that followed the contour of the foam pores observed in SEM micrographs (magnification 1500X). The area inside each polygon, defined by the contour of each pore, was measured and expressed in μm^2^. The pore size distribution was calculated considering at least 100 pores per micrograph. A 2D porosity (areal fraction of the pores) was calculated as the empty (or void space) as calculated by image analysis of micrographs corresponding to the transverse cuts of foams.

### Cell culture experiments

We conducted mammalian cell culture experiments using different mammalian cell lines in wells containing pieces of zein foam. Cells, including mouse embryonic fibroblasts [NIH 3T3 (ATCC CRL-1658] and two prostate cancer cell lines [22RV1 (ATCC CRL-2505) and DU145 (ATCC HTB-81)], were seeded at 1X10^5^ cells/mL (for cancer cells) or 2X10^5^ cells/mL (for fibroblasts) in 24-well ultra-low attachment culture plates containing thin slices of zein foams. Suspension cultures were maintained in 1 mL of DMEM-F12 (Invitrogen, USA) culture media, supplemented with 5% FBS, and incubated at 37°C in a 5% CO_2_ atmosphere for 14 days. Foams were observed after different culture durations using an Axiovert 200 inverted microscope (Carl Zeiss, Germany) and a Stereo V8 stereoscopic microscope (Carl Zeiss, Germany) at 5, 10, and 20X. In addition, an AXIO CSM 700 confocal microscope (Carl Zeiss, Germany) was used for monitoring prostate cancer cell growth within the pores of the zein foams. Samples were observed at 20X with the confocal microscope. In general, for each sample, more than 100 frames were analyzed in the Z-direction, at a resolution no lower than 1 mm.

In an additional set of experiments, NIH 3T3 embryonic fibroblasts were also cultured in static and continuous mode while attached onto the surface of zein foams. For this purpose, the reaction vessel was a rectangular mini-device with an effective volume of 0.1 mL (2mm height, 5mm width, and 15mm length). This reactor was fabricated from poly-lactic acid using a Cube 2 3D printer (Cubify, 3D Systems Inc., Atlanta, GA; USA). A thin layer of TPZ foam (approximately 1 mm thickness) was placed at the bottom of the device chamber. The chamber was covered with a PDMS lid (less than 1 mm thickness) to facilitate oxygen mass transfer. Cells were seeded at a concentration of 1X10^6^ cells/mL in DMEM-F12 (Invitrogen, USA) culture medium supplemented with 5% FBS, and maintained in static incubation at 37°C in a 5% CO_2_ atmosphere; the culture medium was changed every 24 hours. After 120 hours of static culture, samples of the fibroblast cultures on zein foams were inspected using a confocal microscope LSM10 (Carl Zeiss, Germany). For this purpose, samples were fixed in formaldehyde (3.7% formaldehyde in PBS) and 40 μm sections were obtained using a microtome. DAPI (Sigma-Aldrich, Cat. d8417) and MitoTracker Red CM-H_2_Xros (Life Technologies, M-7513) co-staining was used to identify cell nuclei and mitochondria of active cells, respectively.

In additional experiments, after 96 hours of static incubation, DMEM-F12 (Invitrogen, USA) culture media supplemented with 10% FBS and 1% of penicillin-streptomycin was continuously fed using a Fusion 200 classic syringe pump (Chemyx Inc., USA) at a flow rate of 3 μL/min through an inlet port located at one extreme of the chamber. Glucose concentration was determined at the inlet and outlet of the continuous flow chamber using an Accu-Chek Active System (from Roche, Cat. No. 5923786001, USA). The rate of glucose consumption was calculated by dividing the glucose consumption (the difference between the inlet and outlet glucose concentration at steady state) and the effective residence time within the device (calculated as the effective volume over the flow rate = 100 μL/3 μL min^-1^).

## Results and Discussion

We produced a variety of maize-derived plastics, according to the scheme and extrusion protocols described in Materials and Methods (Fig 1; Table 1; Fig 2A and 2B) and exposed them to supercritical CO_2_ foaming (also described in Materials and Methods; Fig 2C and 2D).

We found that the use of either the starch or the main protein fraction of the grain (zein) results in highly porous foams when an adequate plasticizer is chosen. The use of whole maize flour, which is a mixture of corn starch and zein, produces thermoplastic materials that are poorly suitable for supercritical CO_2_ foaming. In the following sections, we discuss these results in detail. In addition, we explore the use of zein foams in mammalian cell culture applications.

### Fabrication of maize-derived thermoplastic materials

Maize-derived thermoplastics were produced under different experimental conditions: variables included (a) the temperature and speed of extrusion, (b) the use of the whole flour, only the starch fraction, only the flour protein fraction, or a mixture of starch and protein, and (c) the use of three plasticizers (sorbitol/glycerol, urea/formamide, and PEG 400 (poly-ethylene glycol with an average molecular weight of 400 Da).

Our experimental design was not exhaustive for all of the possible combinations of materials, plasticizers, and process conditions (see Fig 1; Table 1). For example, we thermo-extruded starch using sorbitol/glycerol and urea/formamide, which are plasticizer systems recommended and used previously for obtaining starch thermoplastics due to their small molecular size and their polar nature. Zein is a more hydrophobic molecule, so we used PEG400. Previous work from our group and others have reported that PEG400 was a suitable plasticizer for zein in casting and thermo-extrusion applications [36,42,44,48]. For mixtures of starch and zein, we used sorbitol/glycerol or urea/formamide to thermoplasticize starch alone and PEG400 to thermoplasticize zein alone. The separately thermoplasticized zein and starch were then co-extruded to create the mixture.

The temperature, speed of screw rotation, and residence time within the extruder are arguably the most relevant parameters of the process conditions that determine the mechanical properties of thermo-extruded materials [49–51]. In our experiments using zein and starch, we established temperature and speed of screw rotation conditions for each material based on experiments reported elsewhere [42]. We selected conditions (Fig 1; Table 1) that yielded plastic materials with adequate mechanical characteristics, as clarified below. The extrusion experiments using whole maize and chemically modified maize flour were conducted at a speed of screw rotation value of 60 rpm based on previous knowledge and we explored a range of temperatures between 100 and 140°C [42,52]. In experiments reported elsewhere, better mechanical properties were found for plastics made from blue maize flours thermo-extruded using sorbitol-glycerol at 60 rpm than at lower or higher rpm values [42,52]. The toughness (area under the curve in a strain vs. stress curve obtained from a mechanical test) was greater for plastics obtained at 120°C than for materials processed at higher or lower temperatures; however, the maximum elongation of these plastics (strain at break) was observed at a processing temperature of 130°C.

### Macroscopic evaluation of foamability of maize derived materials

**Fig 3:**
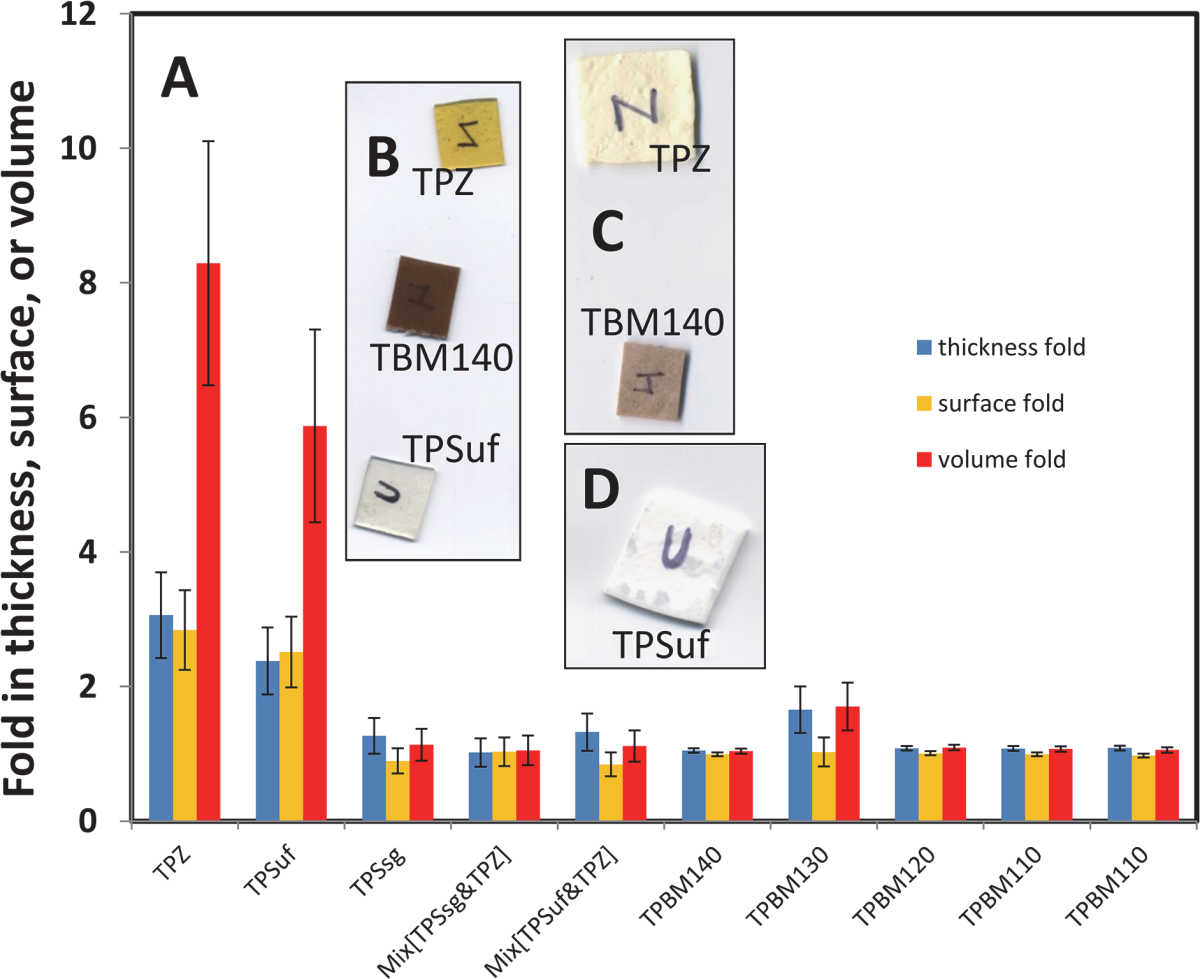
(A) Samples of maize-derived thermoplastics exposed to supercritical CO_2_ foaming exhibited different fold increases in thickness (blue), surface area (yellow), and volume (red). Insets show photographic images of samples elaborated from TPS, TPZ, and TBM140 (B) before and (C,D) after exposure supercritical CO_2_ foaming.

Fig 3A summarizes the results of expansion in the z-direction (thickness increase), in the x-y plane (upper surface expansion), and as a result, in the volume of samples of all the different materials subjected to supercritical CO_2_ foaming in this study. Expansions are expressed as ratios of the values of thickness, upper surface, and volume before and after exposure to foaming conditions. Note that the volumes of the TPZ (thermoplasticized zein slab) and TPSuf (starch slab thermoplasticized with urea/formamide) materials expanded by more than 8 and 6 fold, respectively (Fig 3B–3D). TPBM130 (blue maize thermo-plasticized at 130°C) expanded only modestly (60 to 70%). Interestingly, this material increased in thicknesses but not in x-y surface area (Fig 3B and 3C). TPSsg (starch slabs thermoplasticized with sorbitol-glycerol) materials underwent an increase in volume in the range of 13 to 15%. The rest of the materials showed practically no expansion in the x-y plane (see also Fig 3) and overall volume increments of less than 11%. For TPZ and TPSuf foams, the calculated porosity values (expressed as void fraction) were 0.85+/-0.10 and 0.88+/-0.09, respectively. These values fall within the upper range of porosities reported in literature (50 to 90%) for similar materials [25,33].

### Correlation between strain at break and foaming potential

The supercritical CO_2_ foaming of plastics requires that CO_2_ diffuses into the material (Fig 2C) at supercritical conditions. The amount absorbed depends on the solubilization conditions (temperature and pressure) and solubilization time. At a subsequent stage, a sudden release of pressure triggers CO_2_ expansion, generating bubbles within the material (Fig 2D).

**Fig 4:**
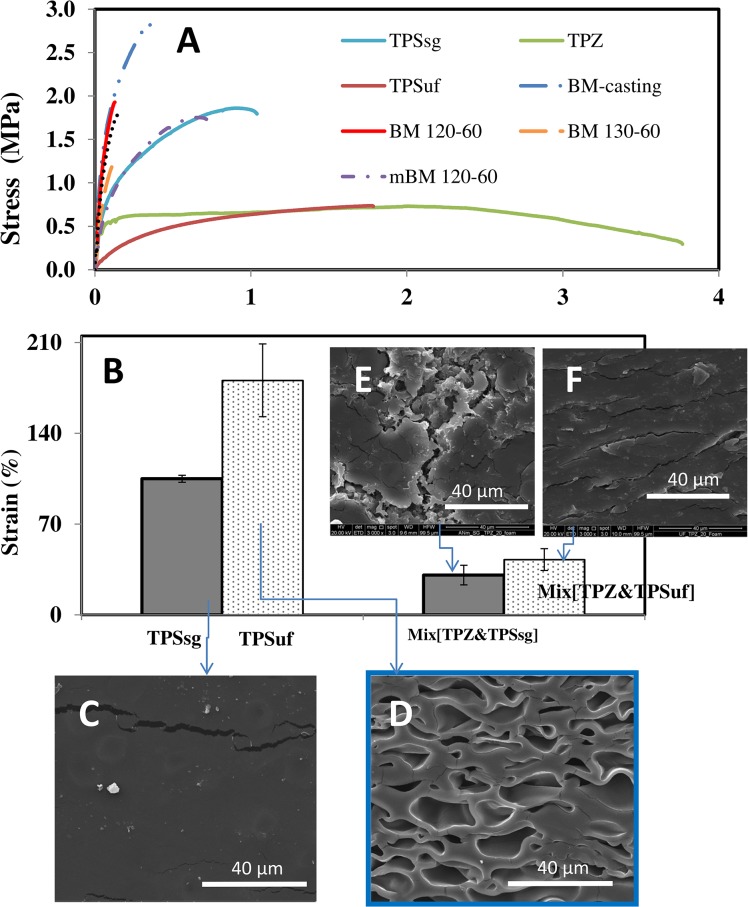
Correlation between elongation and foaming. (A) Strain vs. stress curves for slabs elaborated from different cereal based materials. (B) Effect of different plasticizers on the strain at break of thermoplasticized slabs made from starch (TPS) and TPS/TPZ blends (Mix[TPZ&TPSy]). Plasticizers used were sorbitol/glycerol (red bars), and urea/formamide (blue bars). Mix[TPZ&TPSy] refers to a process in which starch is thermoplasticized with sg (sorbitol-glycerol) or uf (urea-formamide) and then blended with 20% thermoplasticized zein using PEG 400 as plasticizer. Error bars represent the standard deviations of at least 5 replicates. SEM micrographs of maize starch thermoplastics after CO_2_ supercritical foaming: (C) TPSsg, and (D) TPSuf slabs at 3000X. SEM micrographs of thermoplasticized blends of zein and starch thermoplastics after CO_2_ supercritical foaming: (E) Mix[TPZ&TPSsg], and (F) Mix[TPZ&TPSuf] slabs at (3000X).

The likelihood of forming and stabilizing foams therefore depends on numerous factors including the interfacial, rheological, and thermal mass transport properties as well as the melt strength of the expanding matter [17,34]. In particular, the melt strength refers to the extent of deformability and the stress exerted by the polymer among the growing bubbles when subjected to a significant extensional deformation. We indirectly evaluated this by conducting strain and stress testing experiments of the materials studied here using a universal testing machine. In general, strain at break and foamability (i.e., volume expansion as a result of foaming) appeared to correlate reasonably well among the range of samples tested. In our stress vs. strain assays, the strain resistance was clearly higher for TPZ and TPSuf slabs than for materials with lower foaming capacity (Fig 4A and 4B). High elongation potential appears to be a necessary requirement for foaming [35,36].

In our experiments, we produced thermoplasticized slabs from starch using two different plasticizers: sorbitol/glycerol, and urea/formamide. Fig 4B shows a comparison of strain at break of starch materials plasticized with either sorbitol/glycerol or urea/formamide and indicates that the use of urea/formamide as plasticizer enhances strain at break of starch slabs. In terms of strain at break, TPSsg ranked third among all materials tested, but had a strain at break value significantly lower than TPSuf and TPZ. Consistently, when exposed to supercritical foaming conditions, slabs of thermoplasticized starch plasticized with sorbitol/glycerol (TPSsg) underwent little expansion—less than 14% (Fig 3A). The resulting microstructure was practically featureless (Fig 4C), with no evidence of CO_2_ bubbles. In contrast, slabs of starch thermoplasticized with urea/formamide (TPSuf) were successfully foamed under equivalent conditions (Fig 4D), and underwent a more than 6-fold increase in volume.

### Incompatibility in zein and starch mixtures interferes with foaming

**Fig 5:**
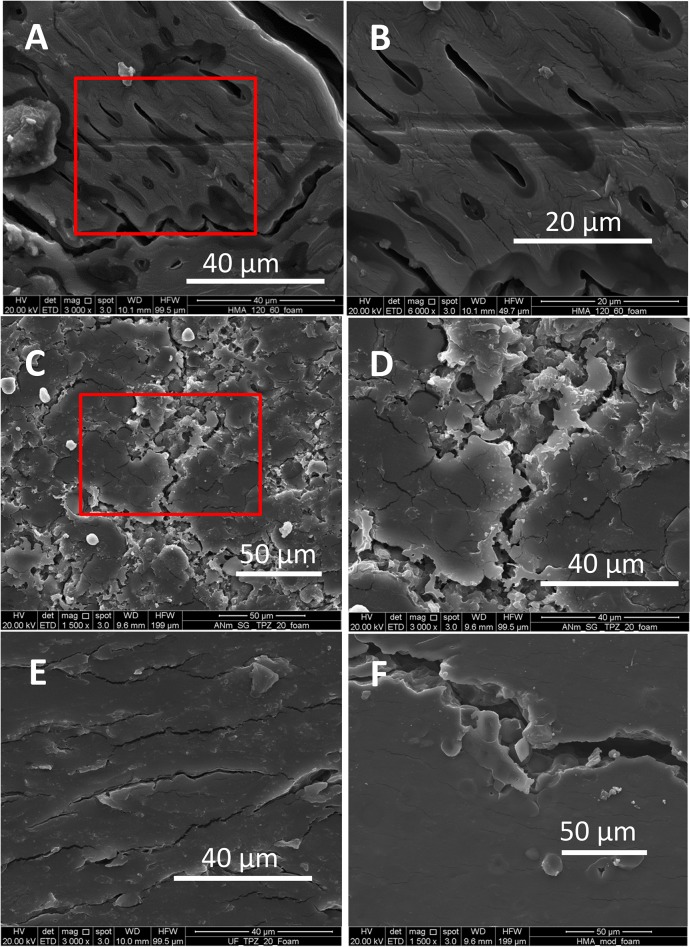
Scanning electronic microscope (SEM) micrographs of transverse cuts of slabs made from whole flour or starch/zein blends after exposure to CO_2_ supercritical foaming: (A) TPBM120 slab (3000X magnification); (B) TPBM120 slab (6000X magnification); (C) Mix[TPZ&TPSsg] slab (1500X magnification); (D) Mix[TPZ&TPSsg] slab (3000X magnification), (E) Mix[TPZ&TPSuf] slab (3000X magnification), and (F) TPmBM slab (1500X magnification).

The addition of zein to the starch system dramatically decreased the strain at break (Fig 4B). Indeed, we were unable to obtain foams from starch/zein mixes (Fig 4E and 4F and 5). Fig 5 shows SEM micrographs of thermoplasticized materials (after foaming) derived from whole flour materials, chemically modified flours, or starch/zein samples. Among them, the common denominators are the coexistence of zein and starch, and the fact that they did not produce foams.

In our experiments with whole flour materials, we explored the effect of different temperatures on foamability. During thermo-extrusion, two competing phenomena concur: plasticization and decomposition [52]. In general, higher temperatures improve plasticization but may cause decomposition, crosslinking, or other side reactions. Similarly, the elongation capacity of a plastic material may be favored by higher temperatures, but jeopardized by decomposition. Therefore, a temperature should exist where the elongation capacity exhibits a maximum. For the thermoplasticization conditions tested to produce BM flour slabs, only processing at 130°C and 60 rpm generated materials capable of a 70% expansion. Extrusion of TPBM compositions at higher or lower temperatures resulted in slabs with lower expansion potential. The material shown in Fig 5A and 5B, produced by thermoplasticization of whole maize flour at 120°C, exhibits a lamellar structure in which some trapped bubbles can be observed. However, the elongation (strain at break) of whole flour slabs is only modest (Fig 4A); microstructure examination suggests that the material was unable to extend (elongate) properly during foaming or that the foam collapsed after foaming.

Flours are complex mixtures, where the interactions between the main components (starch and proteins) do not necessarily result in a sufficiently homogeneous and deformable network capable of efficient retention of CO_2_. Furthermore, the interface weakens the system and sometimes represents a path for loss of the blowing agent [42]. Some of the images in Fig 5 show fractures or cracks, with distinctive features depending on the material (Fig 5C–5F).

Our results indicate that properly thermoplasticized materials derived from the individual components (either the starch or protein fraction), instead of the whole flour, exhibit a high foaming capacity. Next, we analyzed the foamability of single component materials, either starch or zein.

### Foams from starch and zein: microstructural analysis

We were able to obtain foams from slabs of starch thermoplasticized with urea/formamide (TPSuf). The available literature contains a limited number of reports of successful fabrication of fine-structure foams from starch-based materials [38,39]. To our knowledge, this is the first report of production of starch foams by supercritical CO_2_ foaming.

We also reproducibly obtained foams from zein, the most abundant protein in maize. Zein is classified as a prolamine; prolamines are proteins with a high fraction of proline in their structure and are soluble in ethanol [53–55]. Zein is an interesting protein from the point of view of material science: it is hydrophobic, practically insoluble in water, and suitable for thermo-extrusion at relatively low temperatures [42]. In addition, it is considered suitable for cell culture applications [53].

Several reports have documented the use of zein (or zein blends) to produce foams [24,26,28,33,40]. In particular, the zein/PEG system (matrix component/ plasticizer) used here was explored by our group in the past [25]. Here, we use a set of foaming conditions slightly modified from one reported by Salerno et al. [25] that uses a simpler experimental approach using supercritical CO_2_ exclusively as a foaming agent (instead of CO_2_/N_2_ mixes).

**Fig 6:**
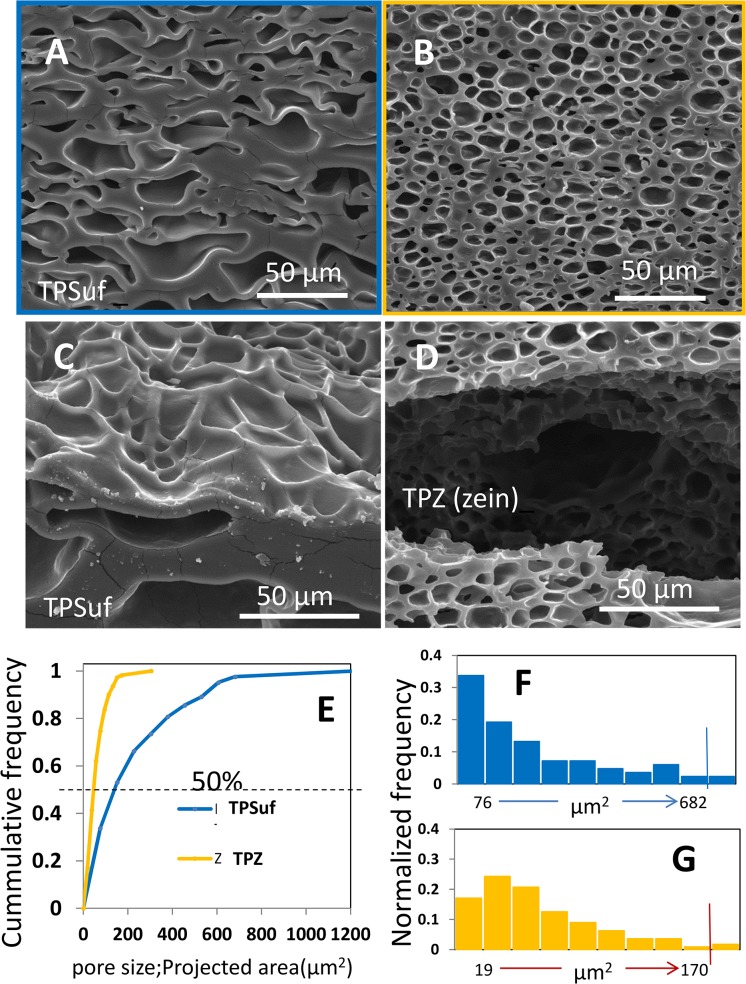
Scanning electronic microscope (SEM) micrographs and pore size distributions of foams. Foams made from (A) starch slabs thermoplasticized at 135°C and 50 rpm using urea/formamide as a plasticizer (sample TPSuf); observed at 1500X, and (B) at 2000X magnification. Foams made from zein slabs thermoplasticized at 75°C and 50 rpm (sample TPZ); observed at (C) 1500X, and (D) 2000X magnification. (E) The cumulative distribution of pore sizes, as calculated by image analysis of SEM micrographs, is presented for TPSuf foams (blue line) and Z foams (yellow line). Pore sizes are expressed in terms of projected areas ([=] μm^2^). The frequency distribution of pore sizes calculated by image analysis of SEM micrographs is presented for (F) TPSuf foams, and (G) TPZ foams.

We conducted a more detailed characterization of the microstructure of selected foams obtained from thermoplasticized zein (TPZ) and thermoplasticized starch plasticized with urea/formamide (TPSuf) using widely available image analysis techniques. Fig 5 presents an analysis of pore morphology, pore size, and pore size distribution based on image analysis of SEM micrographs. Fig 6A and 6B show SEM micrographs of TPSuf and TPZ derived foams at the same magnification (1000X). Similarly, Fig 6C and 6D show SEM micrographs at a 1500X magnification. Visual inspection reveals several differences: for foams obtained at the same foaming conditions, the average size of the pores appears larger for TPSuf foams than for TPZ foams (Fig 6A and 6B). Moreover, in TPSuf foams, larger pores tend to concentrate in a central band within the foam, while smaller pores dominate in the surface regions (not shown). The pores of TPZ foams appear to be more evenly distributed, although a gradient is still apparent, and larger pores are more frequently found in the vicinity of the upper surface. The differences in microstructure are more evident at higher magnification (compare Fig 6C and 6D). A high porosity and a low average pore size were observed in TPZ foams. In addition, the pores appeared to be more elongated in TPSuf foams than in TPZ foams.

Pore sizes are shown (determined as the projected area of each pore (p_i_) in μm^2^) in Fig 6A and Fig 6B. Since the total area of each micrograph can be estimated, the ratio of the sum of all individual pore areas (∑ p_i_) over the total area of the micrograph (A_image_) provides a robust estimator of the 2D porosity of the microstructure (∑ p_i_/A_image_) at a particular transversal cut. The 2D porosity is almost 50% higher in TPZ foams than in TPSuf foams (0.625 versus 0.442). The data on individual pore sizes were used to construct cumulative distribution plots (Fig 5E). Note that in TPZ foams, 50% of the pores are smaller than 50 μm^2^ and practically all pores are smaller than 300 μm^2^. In contrast, in TPSuf foams, the pore distribution is much broader; and 50% of the pores are larger than 150 μm^2^. Fig 6F and 6G present the distributions of pore sizes for TPZ and TPSuf foams, respectively, in normalized histograms. Note that the span (much wider for TPSuf foams) and the shape of the distributions differ substantially. Pore sizes are more evenly distributed for TPZ foams, where smaller pore sizes dominate. The TPSuf foams show a wider span of pore size values. Table 2 summarizes important features of both foam systems: porosity, average pore size, median of pore size, and the standard deviation of pore size. These characteristics confirm that the TPZ foams have a more compact distribution of pore sizes (lower standard deviation) that is dominated by pores of shorter lengths.

### Cell culture experiments in zein foams

**Fig 7:**
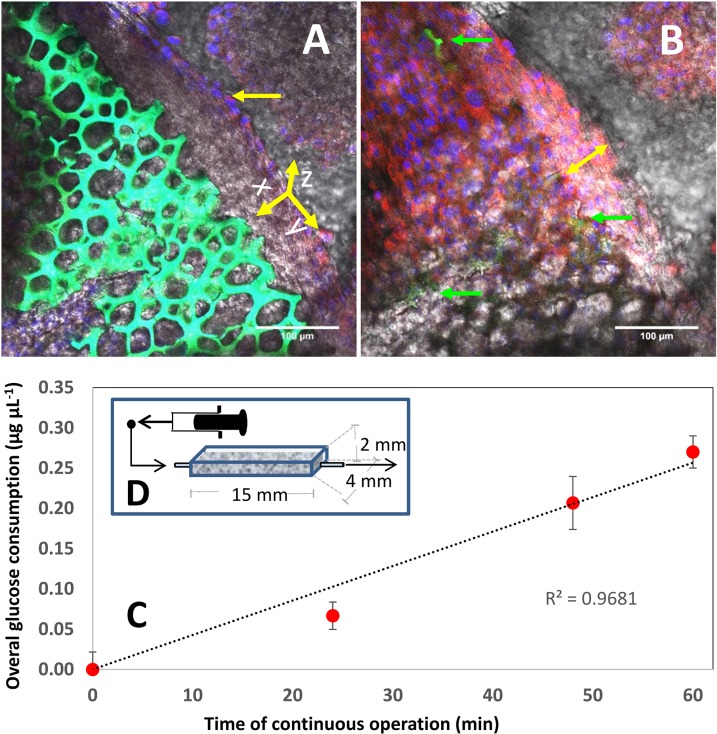
Fibroblasts anchor and proliferate on zein foam surfaces. Confocal microscopy images showing DAPI and MitoTracker co-stained fibroblasts growing on zein foam surfaces. (A) Confocal image showing the intrinsic fluorescence of a zein foam surface (green fluorescent x-y plane). A line of cells (indicated by a single-head arrow), which developed on the edge of the x-y plane, can be seen (blue cell nuclei and red-stained mitochondria). (B) A confocal 2D cut at a slightly closer x-y plane (above that shown in A) reveals the presence of active cells covering the zein surface. DAPI and MitoTracker staining was used to reveal cell nuclei (blue) and mitochondria of metabolically active cells (red). A thick multi-layer of cells is indicated with a double-headed arrow. Green fluorescent regions, corresponding to the zein foams underneath the layer of cells, can be observed (indicated with green arrows). (C) Fibroblasts seeded on zein foams and fed continuously at 0.003 mL min^-1^ in a flow chamber linearly increased their global glucose consumption during the first 60 h of continuous culture. Dotted line indicates best linear fit. Error bars indicate standard deviation of three independent replicates. (D) Scheme of the continuous flow chamber (effective volume of 100 μL).

Starch and zein foams may have many potential applications; here, we present a preliminary exploration of one that is of particular interest to our research group, namely, mammalian cell culture. Cell growth inside the porous structure of foams requires sufficiently wide pores to accommodate cell colonies. Therefore, the average pore size and the distribution of pore sizes of the TPZ foam presented in Fig 6 were not suitable for growth of cell colonies within the porous structure. Modification of the pressure conditions during CO_2_ foaming (see Materials and Methods) allowed us to fabricate materials that had a wider average pore size (wpTPZ foams). Our experiments demonstrate that zein foams can be used as a surface for growth of mammalian cells. We successfully cultured mouse fibroblasts on the surface of wpTPZ foams. Under static conditions, cultures were sustained for 5 days. Fig 7 shows confocal microscopy images of cells attached and growing on the surface of regular polystyrene 96-well plate surfaces (positive control; Fig 7A and 7B), and on thin slices of zein foams. DAPI and MitoTracker staining was used to observe nuclei and mitochondria in metabolically active cells, respectively (Fig 7C and 7D). Layers of cells could be observed in some locations of the foam slices after 5 days of static culture (Fig 7C and 7D). The foam culture system made observation of cells particularly challenging due to the complex 3D topology of the scaffolds. In addition, the intrinsic fluorescence of zein precluded the use of several conventional stains based on green fluorescence emission, such as Live and Dead.

We also conducted experiments where fibroblasts were incubated on zein foams under static conditions for 96 hours in a culture chamber (to allow for proper cell colonization). We then activated a continuous flow of culture medium (at 3μL/min) for an additional period of 96 hours. We monitored the metabolic activity of the culture by quantifying glucose consumption during the first 60 h of continuous perfusion culture. During this period, glucose consumption increased linearly (Fig 7E), reaching 0.270 +/- 0.020 mg/mL after 60 hours of continuous perfusion at a flow rate of 0.003 mL/min. The effective volume of the culture chamber was 100μL (Fig 7F); therefore, the residence time within the device was 33.33 minutes and the glucose consumption rate at 60 h of culture was 8.105 +/- 0.705 μg mL^-1^ min^-1^. This increase in substrate consumption during the first stage of continuous culture suggests that the density of metabolically active fibroblasts within the devices also increased in this time period. In these continuous culture experiments, the outlet stream contained a low cell count (fewer than 1X10^3^cells/mL). Taken together, our results suggest that the cells proliferated and maintained metabolic activity during continuous cultivation on zein foams.

**Fig 8:**
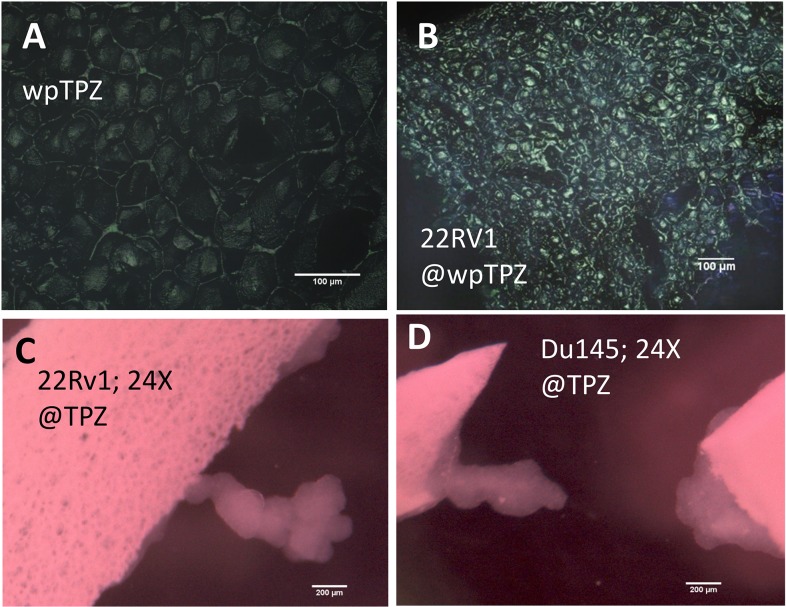
Two different prostate cancer cell lines attach and proliferate on zein foams. A portion of the porous surface of a wpTPZ foam as observed by confocal microscopy at 20X (A) before cell seeding, and (B) 22RV1 cells after seven days of growth. Prostate cancer cell lines cultured on wpTPZ attach, proliferate, and develop into tree-like structures on the edge of wpTPZ foams: (C) 22RV1 cells observed at the third day of culture (24X; stereoscopic microscope); (D) Du145 cells observed at the third day of culture (24X; stereoscopic microscope).

Fig 8 shows images from cell culture experiments were conducted using two different commercially available prostate cancer cell lines: 22RV1 and DU145. These cell lines were able to grow and proliferate on zein foams but not on starch foams or films. We observed different types of colony growth: spread monolayers or multilayer cell structures. Confocal microscopy revealed the proliferation of 22RV1 cancer cells within the exposed pores. Fig 8 shows the structure of these wider pore size foams, as observed using confocal microscopy before (Fig 8A) and after cell proliferation (Fig 8B). Cancer cells populated the interior of the exposed porous structure and essentially achieved confluence after 7 days of culture. Other colonies developed flexible “tree-like” 3D structures able to tolerate the action of slow flow fields (Fig 8C and 8D). These complex structures possibly originate from the ability of these cells to grow both in flat monolayers attached to a substrate, as well as in anchorage-independent conditions in sphere-shaped colonies.

Highly porous plant derived materials, such as the foams studied here, might be a simpler (safer) alternative to matrixes of animal origin for the anchorage culture of mammalian cells; they would enable the establishment of cell multilayer films for tissue engineering applications and of 3-D tumor-like growths for cancer research [56,57].

## Conclusions

In this contribution, maize based materials were assayed to produce bio-foams using supercritical CO_2_ foaming. The foaming potential of an extensive list of materials was assayed, including slabs elaborated from whole flour, only starch, or only the protein fraction (zein) of maize. Materials obtained by casting or thermoplasticization were tested and several variants of thermo compounding conditions were examined. Variation included thermoplasticization at different temperatures, and the use of two different plasticizer systems (sorbitol/glycerol and urea/formamide). The use of either the starch or the main protein fraction of the grain (zein) resulted in highly porous foams when an adequate plasticizer was chosen. The use of whole maize flour or a mixture of corn starch and zein produces thermoplastic materials that are poorly suitable for supercritical CO_2_ foaming.

The tested set of experimental conditions and materials revealed that TPSuf and TPZ slabs rendered the best foams. Remarkably, these materials experienced a volume expansion of more than 6-fold. A detailed characterization of the microstructure of these materials through image analysis of SEM micrographs revealed that TPZ and TPSuf foams differ in terms of overall porosity, average pore size, pore size distribution, and pore morphology. TPZ foams are more porous and their size distribution is more evenly distributed among a narrower span of pore sizes when compared to TPSuf foams.

We also observed a correlation between strain at break and foaming potential of a material. Therefore, the foaming potential of a material can be preliminarily evaluated from stress vs. strain rate data. Conversely, the foaming capacity (or capacity or adequacy to produce foams) can be used for indirect assessment of the strength and flexibility of a material. Our results also suggest that foaming capacity can be a useful indicator of the strain resistance and flexibility of these types of biomaterials.

The results from proof-of-concept mammalian cell culture experiments conducted on the surface of thermoplasticized zein foams suggest that these surfaces support cell proliferation. Four types of mammalian cells—mouse and human fibroblasts and two different prostate cancer cell lines (22RV1 and DU145)—were able to attach and proliferate on thermoplasticized zein foams.
